# Targeting persistently activated inflammatory microenvironment to promote chronic wound healing

**DOI:** 10.3389/fimmu.2025.1708358

**Published:** 2025-12-17

**Authors:** Yuxuan Dai, Yu Chen

**Affiliations:** 1 Department of Plastic Surgery, Beilun Branch of the First Affiliated Hospital, College of Medicine, Zhejiang University, Ningbo, Zhejiang, China; 2 Department of Surgical Oncology, The First Affiliated Hospital, School of Medicine, Zhejiang University, Hangzhou, Zhejiang, China

**Keywords:** tissue repair, delayed healing wounds, macrophages, inflammation, immunoregulation

## Abstract

The immune system plays a pivotal role in maintaining the balance of inflammatory responses and facilitating tissue repair and wound healing. However, under the combined influence of immune microenvironmental factors and external stimuli, immune cell dysfunction can lead to persistent activation of the inflammatory milieu, resulting in delayed or impaired wound healing. Therefore, regulating immune responses within the chronic inflammatory microenvironment and suppressing aberrant immune cell activation not only helps restore immune homeostasis but also effectively accelerates the wound healing process. Identifying and modulating novel targets associated with macrophage and T-cell dysregulation, as well as the crosstalk among immune cells, offers new insights and therapeutic strategies for the treatment of chronic inflammation-related disorders and wound repair. This review focuses on the molecular mechanisms underlying aberrant macrophage and T-cell activation, the intercellular crosstalk within the immune microenvironment, and their impact on the wound healing process. Furthermore, it highlights potential therapeutic targets for limiting persistent inflammation and re-establishing immune homeostasis. Elucidating these mechanisms and targets may provide promising avenues for the treatment of chronic inflammatory diseases and for advancing strategies in tissue repair and regeneration.

## 1 Introduction

Delayed healing and non-healing of chronic wounds represent a class of clinical problems driven by the persistent activation of the chronic inflammatory microenvironment, a process that involves multiple levels of immune regulation (1). The local inflammatory milieu plays a critical role in protecting the host against infection, repairing tissue damage, and maintaining systemic homeostasis. Effective interactions between immune cells and their specific ligands form the basis of immune function (2, 3). However, under the combined influence of the immune microenvironment and environmental stimuli, immune dysfunction may occur, leading to abnormal activation of the inflammatory milieu and thereby contributing to impaired or delayed wound healing (4, 5). Chronic non-healing wounds are typically characterized by sustained production of inflammatory mediators and cytokines, along with aberrant activation of macrophages and T cells. These abnormalities suppress the normal wound healing process, resulting in prolonged tissue damage and functional impairment (6).

During chronic wound healing, dysregulation of the immune system is one of the major causes of impaired or non-healing wounds (7, 8). Wound healing is classically divided into several overlapping phases, including inflammation, proliferation, and repair (9). Under physiological conditions, macrophages play a key role in the early inflammatory phase by clearing bacteria and cellular debris and by initiating tissue repair, whereas T cells are primarily involved in the subsequent adaptive immune response (10, 11). Macrophages are critical effector cells of the innate immune system that participate in pathogen recognition, antigen presentation, tissue repair, and the maintenance of immune homeostasis (12). A defining feature of macrophages is their high degree of plasticity, whereby they rapidly adjust their phenotype, metabolic state, and functional profile in response to distinct microenvironmental cues to meet the demands of inflammation, tissue remodeling, or homeostasis (13). This plasticity is not limited to the classical M1/M2 dichotomy, but rather reflects a dynamically regulated continuum of activation states, constituting a central regulatory hub in diverse pathological contexts such as chronic inflammation, the tumor microenvironment, and tissue regeneration. Accumulating evidence indicates that macrophages do not exist in fixed M1 or M2 states; instead, they exhibit a continuous and reversible spectrum of functional programs driven by various combinations of signals, including Interleukin-6 (IL-6)/Transforming Growth Factor-beta (TGF-β),Granulocyte-Macrophage Colony-Stimulating Factor (GM-CSF)/Macrophage Colony-Stimulating Factor (M-CSF), lipid mediators, and hypoxic conditions (14, 15). For example, in the tumor microenvironment, tumor-associated macrophages (TAMs) can concomitantly express both M1 and M2 associated markers, whereas during wound repair, the transition from an M1-like to an M2-like phenotype is a critical step for resolution of inflammation and initiation of regeneration (16, 17). Failure of macrophages to switch effectively from a pro-inflammatory to a pro-reparative phenotype leads to sustained activation of M1-like macrophages, while aberrant interactions between dendritic cells (DCs) and T cells further drive persistent immune activation (18, 19). In particular, dysregulated activation of macrophages, T helper 1 (Th1) cells and T helper 17 (Th17) cells contributes to continuous delay of the wound healing process (20). Classically activated M1 macrophages promote inflammatory responses during the early stages of wound healing, whereas alternatively activated M2 macrophages exert anti-inflammatory and pro-repair functions during the later healing phase (21, 22). However, within a chronic inflammatory microenvironment, excessive activation of M1 macrophages results in a prolonged inflammatory state, suppresses the functions of M2 macrophages, and ultimately impedes the progression of wound healing (23, 24).

In addition, aberrant activation of Th1 and Th17 cells particularly under dysregulated control of the co-stimulatory molecule Inducible T-cell COStimulator (ICOS) and Cytotoxic T-Lymphocyte Antigen 4 (CTLA-4) further exacerbates immune imbalance and contributes to the non-healing state of chronic wounds (25, 26). In the context of the dysregulated immune microenvironment associated with impaired chronic wound healing, targeting the abnormal activation of immune cells and their receptors or downstream signaling pathways has emerged as a promising therapeutic strategy (27, 28). By modulating receptor function or intracellular signaling in immune cells, it is possible to effectively suppress pathological activation of macrophages, Th1 cells, and Th17 cells, thereby restoring immune homeostasis and promoting the normal progression of wound repair (29, 30). Targeting the aberrant crosstalk between macrophages and T cells and attenuating persistently activated inflammatory responses may represent a key approach to improving chronic wound healing outcomes (31, 32). Therefore, therapeutically modulating the persistently activated inflammatory microenvironment with a particular focus on regulating macrophage and T-cell function has become a potential new strategy for enhancing tissue repair and promoting the healing of chronic wounds.

## 2 Molecular mechanisms of normal wound healing

Wound healing is a complex, highly coordinated process involving multiple molecular mechanisms, which are generally divided into four major, overlapping phases: hemostasis, inflammation, proliferation, and remodeling. Among these, the proliferative phase is a critical stage, primarily characterized by cell proliferation, angiogenesis, and extracellular matrix (ECM) remodeling (33, 34). During this phase, multiple molecular signaling pathways are engaged, and in particular, the regulatory mechanisms governing cell proliferation are essential for the subsequent progression of wound repair (9, 18).

### 2.1 Process of normal wound healing

When tissue injury occurs, the first response is hemostasis, which is achieved through vasoconstriction, platelet aggregation, and activation of coagulation factors. Platelets release a variety of growth factors, such as Platelet-Derived Growth Factor (PDGF), TGF-β, and Vascular endothelial growth factor (VEGF), all of which play important roles throughout the wound-healing process (35). The primary function of the inflammatory phase is to eliminate pathogens and remove damaged cells from the wound site. Innate immune cells, including neutrophils and macrophages, initiate the inflammatory response by secreting cytokines such as IL-1 and Tumor Necrosis Factor-alpha (TNF-α). These cytokines not only contribute to pathogen clearance but also provide essential signals for the transition to the proliferative phase (36). The hallmark of the proliferative phase is the proliferation of fibroblasts, epithelial cells, endothelial cells, and other cell types. Growth factors such as TGF-β, Epidermal Growth Factor (EGF) and Fibroblast Growth Factor (FGF) are key regulators of the proliferation of these cells. By binding to their cognate receptors on the cell surface, they activate downstream signaling pathways including Mitogen-Activated Protein Kinase (MAPK) and Phosphatidylinositol 3-Kinase (PI3K)-Akt, thereby promoting cell proliferation and migration (37). VEGF plays a pivotal role in angiogenesis during the proliferative phase. VEGF stimulates endothelial cell proliferation and migration, promoting the formation of new blood vessels and thereby supplying the wound area with oxygen and nutrients required for repair (38). ECM remodeling is another crucial component of the proliferative phase. Fibroblasts synthesize large amounts of ECM components, including collagen and elastin, and regulate ECM degradation and reconstruction via matrix metalloproteinases (MMPs) and their tissue inhibitors (TIMPs) (39, 40). During the remodeling phase, the structural organization of the wound gradually approaches that of normal tissue. Collagen fibers become more regularly aligned, and the tensile strength of the wound progressively increases. Fibroblasts further differentiate into myofibroblasts, which contribute to wound contraction and closure. The molecular mechanisms involved at this stage include regulation of fibroblast function by TGF-β, IL-10, and other mediators (41).

### 2.2 Influence of molecular mechanisms on the proliferative phase

Throughout wound healing, multiple signaling pathways act in a coordinated manner to promote cell proliferation and tissue repair. First, in terms of growth factor–receptor interactions, TGF-β plays a dual role in wound healing: it promotes cell proliferation while also regulating fibroblast migration and collagen synthesis. However, excessive TGF-β activation may lead to fibrosis and adversely affect the quality of wound repair (42). EGF and FGF promote the proliferation of keratinocytes and fibroblasts during the early stages of wound healing, thereby facilitating re-epithelialization and wound closure. EGF primarily enhances cell proliferation via activation of the Extracellular Signal-Regulated Kinase (ERK) signaling pathway, whereas FGF contributes to angiogenesis and ECM remodeling through activation of MAPK, PI3K/Akt, and other pathways (42, 43). Second, at the level of intracellular signaling, the PI3K/Akt pathway supports wound repair by promoting cell survival, proliferation, and migration. During the proliferative phase, Akt accelerates the healing process by enhancing the growth of fibroblasts and endothelial cells. The MAPK pathway is also crucial for cell proliferation, migration, differentiation, and stress responses, particularly in driving the proliferation of epithelial cells and fibroblasts after injury (44). Third, with regard to ECM remodeling, fibroblast activity during the proliferative phase is a key determinant of successful wound healing. MMPs mediate degradation of excessive or damaged ECM components, thereby facilitating the formation of new tissue. However, excessive matrix degradation or incomplete reconstruction can result in scar formation or hypertrophic scarring (45). Finally, immune cells also play an important role in this phase. Macrophages contribute to the proliferative phase by secreting cytokines such as TGF-β and IL-10, which promote fibroblast proliferation and collagen synthesis. In addition, macrophages assist wound repair by clearing necrotic cells and residual pathogens (46, 47).

Overall, the proliferative phase of wound healing is characterized by the convergence of multiple processes, including cell proliferation, angiogenesis, and ECM remodeling. Molecular mediators such as TGF-β, VEGF, EGF, and FGF exert crucial functions during this stage by regulating cell proliferation and migration, thereby driving wound repair and closure (48). The coordinated action of these mechanisms not only determines the efficiency of wound healing but also profoundly influences the final outcome, including scar formation and the overall quality of tissue repair.

## 3 Mechanisms of delayed or non-healing chronic wounds

**Figure 1:**
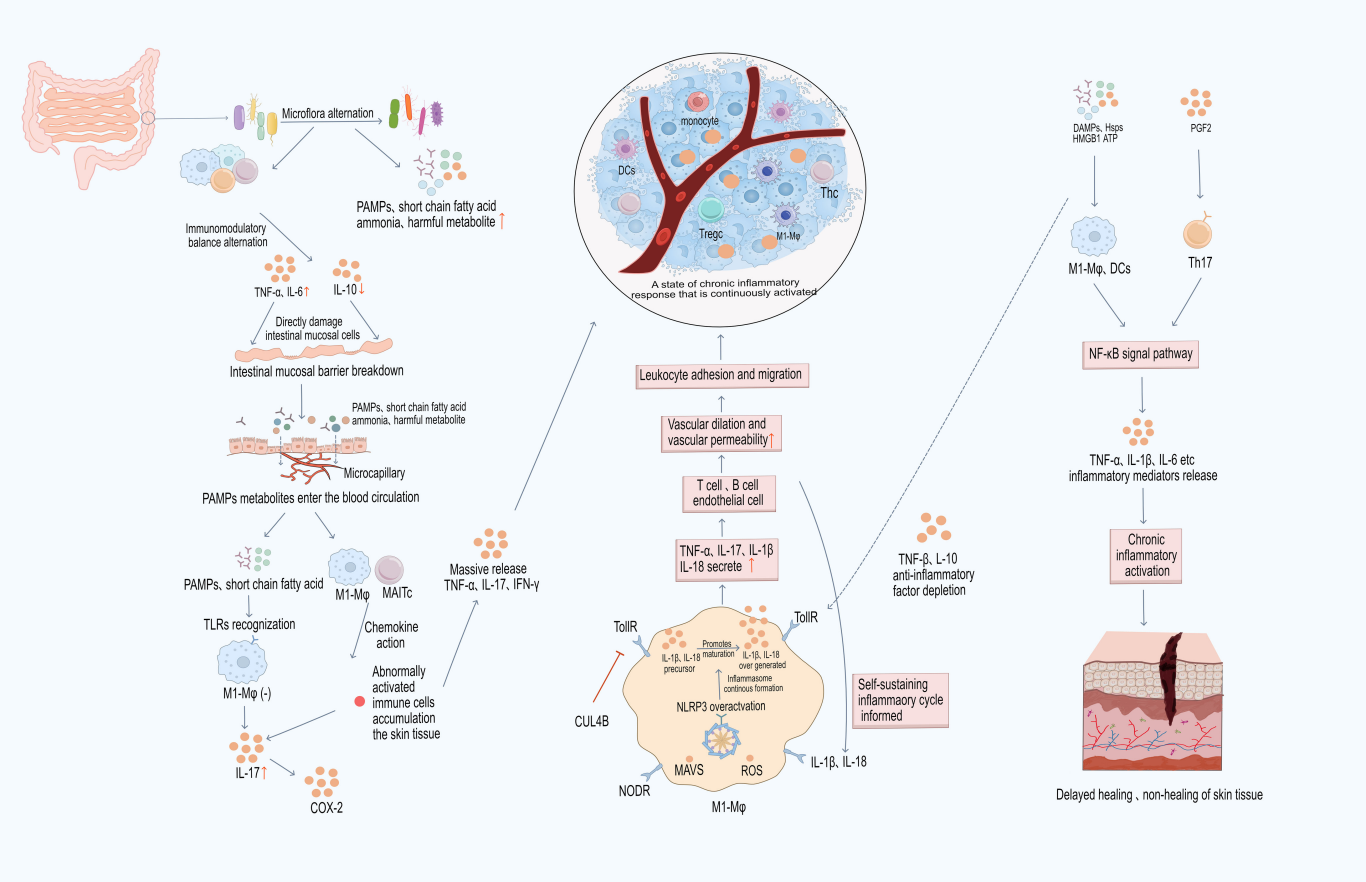
Specific molecular mechanisms driving sustained activation in delayed or non-healing skin tissue. During infection, PAMPs such as LPS are recognized by PRRs on M1 macrophages and dendritic cells. In macrophages, ATP binds to the P2X7 receptor, leading to membrane depolarization and potassium ion efflux, which fully activates the NLRP3 inflammasome. ROS can also promote NLRP3 activation through oxidative modification and alteration of the intracellular environment. The activated NLRP3 inflammasome drives the release of IL-1β and IL-18, which are primarily secreted by M1 macrophages and act on IL-1β and IL-18 receptors through autocrine/paracrine signaling. This amplifies macrophage activation and prolongs the inflammatory phase. IL-1β and IL-18 also act on other immune cells, further exacerbating the local inflammatory response. Activated M1 macrophages secrete IFN-γ and IL-2, recruiting more immune cells to the wound, leading to sustained neutrophil infiltration, accumulation of proteases and ROS, matrix degradation, and disrupted angiogenesis, which inhibit the function of fibroblasts and keratinocytes, hindering reepithelialization. Gut microbiota dysbiosis allows LPS and SCFAs to cross the intestinal barrier and enter the bloodstream; at the wound site, TLR recognition promotes macrophage polarization toward the M1 phenotype and, through the “gut-skin/wound axis,” enhances the accumulation of MAIT cells, further disrupting the underlying microenvironment and causing delayed or non-healing. Chronic mechanical stress and other sterile injuries release DAMPs (such as HMGB1, ATP), which bind to PRRs on dendritic cells and macrophages, inducing macrophages to express IL-23 and IL-17. IL-12 and IFN-γ drive CD4^+^ T cell differentiation into Th1 cells, further activating macrophages, weakening the suppressive effects of Treg cells, and activating CD8^+^ T cells. This leads to further damage of epithelial cells and fibroblasts at the wound edge, disrupting immune tolerance and repair rhythms. Macrophage-derived IL-23 promotes Th17 cell differentiation, and the key effector molecule IL-17A upregulates COX-2, maintaining PGE_2_ production. This results in vasodilation and leakage, enhancing macrophage recruitment/activation, amplifying the protease/antiprotease imbalance, and increasing oxidative stress. These processes consume growth factors, damage the extracellular matrix and granulation tissue quality, and ultimately lock the wound in a persistent inflammatory state, leading to delayed or non-healing.

One of the fundamental causes of delayed or non-healing chronic wounds is persistent activation of inflammation. Recent studies have shown that endogenous signals such as pathogen-associated molecular patterns (PAMPs) and damage-associated molecular patterns (DAMPs) in the local wound environment, as well as immune cells and cytokines, act as “triggers” for sustained immune activation (49, 50). However, while many studies highlight the role of these factors in the persistence of inflammation, current research often overlooks their potential interactions and the specific mechanisms by which they contribute to chronic wound healing (**Figure 1**). PAMPs and DAMPs interact with immune cell surface receptors to activate downstream immune responses, maintaining a prolonged low-level inflammatory state that prevents the wound from transitioning smoothly from the inflammatory phase to the proliferative and remodeling phases, ultimately leading to tissue repair failure (49, 50). Notably, the delay in chronic wound healing is not merely a result of immune dysregulation but is a consequence of the intertwined effects of multiple biological pathways. These factors act through various cellular signaling pathways, collectively shaping the local immune response and creating a persistent inflammatory environment. While current research has elucidated some mechanisms, the synergistic effects of multiple pathways and their impact on chronic wounds remain underexplored.

### 3.1 Overactivation of inflammatory cells

Normal inflammation is a short-term and controlled physiological process aimed at clearing pathogens and necrotic tissue, thereby creating favorable conditions for subsequent repair. However, in chronic wounds, the inflammatory response often persists for extended periods, leading to prolonged infiltration and overactivation of neutrophils and macrophages. These cells continuously release pro-inflammatory cytokines such as TNF-α, IL-1, and IL-6, thereby exacerbating local inflammation (51, 52). This persistent immune response not only disrupts the wound healing process but also causes continuous damage to the local tissue. Excessive inflammation first manifests as apoptosis and tissue necrosis (53). The over-release of inflammatory mediators induces apoptosis in normal cells at the wound site, such as fibroblasts and endothelial cells, thereby inhibiting their proliferation and migration and further hindering wound repair (36, 54) Additionally, the sustained activation of immune cells leads to their prolonged accumulation at the wound site, depleting the resources necessary for repair. This accumulation may also result in secondary damage to already-repaired tissue, intensifying the vicious cycle of chronic inflammation.

Chronic wound inflammation is closely associated with dysfunctional macrophage activity and excessive activation of the NOD-like Receptor Family Pyrin Domain-Containing 3 (NLRP3) inflammasome in innate immunity (55). The interaction between PAMPs and pattern recognition receptors (PRRs) is considered a key driver in maintaining the active inflammatory state within the microenvironment (56). As such, strategies targeting macrophage polarization and NLRP3 inflammasome activation are becoming a major focus of chronic wound treatment research. At the wound site, bacterial lipopolysaccharides (LPS), viral double-stranded RNA, and other PAMPs can be recognized by Toll-like receptors (TLRs) and NOD-like receptors (NLRs) on macrophages and dendritic cells. This activates the Nuclear Factor kappa-B(NF-κB) and Interferon Regulatory Factor 3 (IRF3) signaling pathways, inducing the expression and release of pro-inflammatory cytokines such as pro-IL-1β, TNF-α, and IL-18 (57, 58). IL-1β and IL-18 further promote the polarization of monocytes to M1 macrophages through autocrine and paracrine signaling, amplifying the inflammatory response (59).

M1 macrophages are one of the primary effector cells of the NLRP3 inflammasome. On top of the initial PAMPs-PRRs signaling, DAMPs such as ATP, reactive oxygen species (ROS), uric acid crystals, and cholesterol crystals further trigger the assembly and full activation of the NLRP3 inflammasome (60, 61). For example, large amounts of ATP released by damaged cells induce potassium ion efflux via the P2X7 receptor, leading to a decrease in intracellular potassium concentrations, which subsequently drives NLRP3 inflammasome activation (62). Simultaneously, ROS plays a crucial regulatory role, potentially promoting the amplification of inflammation by oxidatively modifying key molecules or altering the cellular redox environment. Therefore, strategies targeting P2X7 receptor antagonists or inhibiting ROS production are considered promising for improving chronic wound healing and are being tested in clinical trials to verify their efficacy (63). The activation of the NLRP3 inflammasome results in the conversion of pro-caspase-1 to active caspase-1, which then cleaves pro-IL-1β and pro-IL-18 into their mature forms and releases them (64, 65). These effector molecules not only directly exacerbate inflammation but also enhance M1 macrophage polarization, forming a self-amplifying inflammatory positive feedback loop. This loop drives the continuous release of ATP, ROS, and other molecules into the tissue microenvironment, further activating PRRs and maintaining a persistent “TLR4/macrophage/NLRP3” inflammatory axis. The outcome is tissue necrosis and impaired repair, keeping the wound trapped in the inflammatory phase and preventing progression to the healing phase (66, 67).

While research in this field has provided important insights into the immunological mechanisms of chronic wounds, some studies still have limitations. First, although existing studies have demonstrated the pivotal role of the NLRP3 inflammasome in chronic wound inflammation, the specific molecular mechanisms remain incompletely understood. Many studies focus on isolated mechanisms or molecules, lacking a systematic analysis of their interactions (68). Therefore, more research is needed to unravel the complex interplay between these cytokines and signaling pathways (69). Second, while studies on targeting P2X7 receptors or ROS inhibitors in chronic wound therapy have strong preclinical evidence, the success rate of clinical trials remains low. Some trials suffer from small sample sizes and design flaws, leading to inconclusive results and requiring further verification (70, 71). As such, future research should not only strengthen in-depth analysis in basic research but also focus more on optimizing the design of clinical studies to ensure the efficacy and safety of new therapies.

### 3.2 Abnormal activation of APCs maintains the inflammatory environment of chronic wounds

During normal tissue repair, Regulatory T cells (T_Reg_) constrain excessive activation of Th cells and cytotoxic T lymphocytes (CTLs) to maintain immune homeostasis and a controllable, self-limited inflammatory phase. Mechanistically, T_Reg_ cells highly express CTLA-4, which binds B7 molecules (CD80/CD86) on antigen-presenting cells (APCs) and competitively inhibits CD28-mediated co-stimulation (72, 73). A process essential for timely resolution of early wound inflammation. In parallel, Programmed Death-Ligand 1 (PD-L1) on T cells engages PD-L1/PD-L2 on APCs to deliver inhibitory signals that further restrict T cell activity (74, 75). T_Reg_ -derived IL-10 and TGF-β, together with arginase-1 (ARG1) and inducible nitric oxide synthase (iNOS) from myeloid-derived suppressor cells (MDSCs), suppress effector T-cell overactivation (76, 77). In addition, the CD39/CD73 ectonucleotidase pathway on APCs generates adenosine, which signals through A2A receptors to dampen T-cell function. Collectively, these checkpoints terminate inflammation at an appropriate level and permit progression into tissue repair and remodeling.

In chronic wound settings, however, persistent pathogen products and necrotic tissue components drive presentation of aberrant antigens, breaking immune tolerance and sustaining long-lived activation of APCs and CTLs. Activated APCs continuously stimulate CD4^+^ and CD8^+^ T cells via MHC class II and cross-presentation, and their heightened expression of CD80/CD86 strengthens CD28-dependent co-stimulation, creating a self-reinforcing activation loop (78, 79). Concomitantly, APC-derived TNF-α, IFN-γ, IL-12, and IL-2 promote differentiation of naïve Th0 cells into Th1, Th2, and Th17 subsets, amplifying both cytotoxic and humoral responses (80, 81). Th2 expressed CD40L engages CD40 on B cells, driving robust antibody production and immune complex deposition. These immune complexes are continuously taken up and processed by APCs, sustaining cytokine release and local tissue injury and establishing a hard-to-break positive feedback loop that directly contributes to delayed or non-healing wounds (82, 83). Although CTLA-4 is often transiently upregulated on activated Th1/Th2/Th17 cells and CTLs to restrain responses, this inhibitory pathway is frequently inadequate or dysfunctional in chronic wounds, permitting persistently hyperactive effector T cells that damage tissue (84, 85). Ongoing antigenic stimulation and autoreactive antibodies secreted by plasma cells further augment immune complex formation, exacerbating local necrosis, fibrosis, and microvascular injury and thereby suppressing effective angiogenesis and fibroblast-mediated repair (86, 87). In parallel, macrophages skew toward an M1 phenotype under sustained exposure to IFN-γ and TNF-α, producing excessive inflammatory mediators and ROS, which impedes M2-driven tissue reconstruction and neovascularization (88, 89).

Thus, aberrant activation of APCs together with dysregulated T-cell responses in chronic wounds not only prolongs the inflammatory phase but also blocks the initiation of repair, culminating in an “immune–inflammation lock-in” state. Therapeutically, epigenetic attenuation of excessive CD80/CD86–CD28 interactions or enhancement of negative checkpoints such as CTLA-4 and PD-1 may restore T-cell tolerance and reduce immunopathology (90, 91). Concurrent strategies that redirect macrophage polarization (inhibiting M1, promoting M2) and temper overactive Th1/Th17 responses hold promise for rebalancing the local immune microenvironment, enabling transition from persistent inflammation to tissue repair and ultimately improving chronic wound healing outcomes (92).

Despite current research highlighting various immune regulatory mechanisms in wound healing, these mechanisms are often interwoven and complex. For instance, although CTLA-4 plays a crucial inhibitory role in normal immune responses, its dysregulation in the chronic wound environment may exacerbate immune imbalance (25, 93). Therefore, future studies should further investigate the specific mechanisms underlying CTLA-4 dysfunction in chronic wound healing to better understand its therapeutic potential. While molecules such as IL-10 and TGF-β are key players in the immune regulatory functions of T_Reg_ cells, their dual roles in chronic inflammation—where they can both suppress immune responses and potentially promote fibrosis—highlight the need for more research into their precise mechanisms (94, 95). The roles of these factors may vary across different stages of wound healing, and further studies are necessary to elucidate their specific functions within the local immune environment. Regarding the regulation of macrophage polarization, current research primarily focuses on accelerating wound healing by inhibiting M1 macrophages or promoting M2 macrophage polarization. However, whether macrophage polarization is a singular target or if there are additional subtypes and regulatory pathways yet to be identified remains to be experimentally verified. Further investigation is needed to determine the broader scope of macrophage polarization and its potential impact on chronic wound healing.

### 3.3 Metabolic and oxidative stress imbalance

#### 3.3.1 Imbalance of oxidative stress responses

Persistent inflammation is usually accompanied by high levels of oxidative stress and metabolic dysregulation, which together aggravate cellular damage and profoundly impair normal healing. Continuous generation of free radicals and ROS is a major driving force of cellular injury, further amplifying local inflammation and tissue destruction and thereby suppressing the repair process (96). However, although the detrimental role of free radicals and ROS in cellular damage is widely recognized, some studies have suggested that ROS at low concentrations may exert beneficial effects on cellular repair (97, 98). In addition, sustained inflammatory responses disrupt normal metabolic processes within the wound area, leading to insufficient cellular energy supply. The activity of reparative cells, such as fibroblasts and endothelial cells, declines, which further compromises tissue repair capacity and worsens the healing status of chronic wounds (99). This controversy highlights the dual role of ROS in cellular physiology and underscores the need for more refined, concentration-dependent analyses when investigating their functions.

Persistent oxidative stress impairs cell membranes, DNA, and protein structures, thereby inhibiting cellular proliferation and migration and delaying the course of wound repair (100, 101). It is noteworthy, however, that most existing studies on oxidative stress and wound healing have focused on acute wounds, and there is still no consensus regarding its impact on chronic wounds. This discrepancy may be related to the fact that the long-term microenvironment of chronic wounds differs substantially from that of acute injuries. Future studies should pay greater attention to this distinction in order to elucidate the specific roles of oxidative stress in chronic wound healing (102, 103).

Moreover, sustained inflammation disrupts normal metabolic processes in the wound region, resulting in inadequate energy supply to cells. The reduced activity of reparative cells, including fibroblasts and endothelial cells, further weakens tissue repair capacity and aggravates poor healing in chronic wounds (104, 105). Although this viewpoint is supported by multiple studies, other reports indicate that the decline in reparative cell activity may not be solely attributable to energy deficiency, but is also closely linked to alterations in intracellular signaling pathways (106). Dysregulation of these pathways may play an important role in the wound healing process, suggesting that therapeutic strategies should take into account multidimensional cellular and molecular mechanisms.

#### 3.3.2 Gut microbiota and metabolic imbalance

Dysbiosis of the gut microbiota is closely associated with the pathogenesis of delayed and non-healing chronic wounds. Studies have shown that patients with hard-to-heal chronic wounds often exhibit marked alterations in gut microbial composition, accompanied by impaired intestinal barrier function, leading to a “leaky gut” phenotype. Increased intestinal permeability allows microorganisms and their derived molecules (such as LPS) to translocate across the intestinal wall, enter the circulation, and reach the wound site, where they are recognized by pattern recognition receptors (e.g., TLRs) expressed on locally enriched macrophages and dendritic cells. This aberrant activation of immune cells further amplifies inflammatory responses and aggravates tissue damage (107, 108). Consequently, gut microbiota dysbiosis and disruption of the intestinal barrier jointly drive the persistence of inflammatory responses and the delay of tissue repair in chronic wounds. Although this process is supported by experimental data, the prevalence and precise mechanisms of “leaky gut” across different individuals remain a matter of debate. Some studies suggest that whether gut dysbiosis directly causes delayed chronic wound healing still requires confirmation in larger, well-designed clinical trials.

Another hallmark of gut microbiota dysbiosis is reduced levels of short-chain fatty acids (SCFAs) (109). SCFAs are known to enhance the expression of Phosphatase and Tensin homolog (PTEN), which in turn restricts excessive inflammatory activation by inhibiting the PI3K-Akt signaling pathway (110, 111). Insufficient SCFA availability disrupts this regulatory mechanism, leading to abnormal M1 macrophage polarization and heightened pro-inflammatory activity, thereby further impairing wound healing (112). Simultaneously, SCFA deficiency compromises T-cell regulatory functions, driving excessive activation of effector subsets such as Th1 and Th17 cells, which release large amounts of IFN-γ and IL-17. These cytokines intensify local immune responses and tissue injury (113). The combined abnormal activation of macrophages and T cells establishes a self-perpetuating inflammatory–immune feedback loop that represents a key pathological barrier to wound resolution. In addition to SCFAs, other gut microbiota-derived metabolites (e.g., serotonin) and circulating inflammatory cytokines (e.g., IL-6, TNF-α) modulate peripheral immune homeostasis and indirectly influence cutaneous wound repair (114, 115). Notably, mucosal-associated invariant T (MAIT) cells of gut origin have been found enriched within chronic wound tissues. These cells secrete abundant pro-inflammatory mediators, including IFN-γ, TNF-α, and IL-17, which suppress fibroblast activity and angiogenesis, thereby further impeding tissue regeneration. Thus, therapeutic strategies aimed at restoring gut microbiota composition and metabolite production (e.g., SCFAs and serotonin) to reestablish the balance of M1 macrophage and T-cell activity are considered promising approaches for enhancing chronic wound healing (116, 117).

Accordingly, targeting the sustained activation of the inflammatory microenvironment in chronic wounds—particularly by inhibiting aberrant M1 macrophage polarization, limiting overactivation of Th1/Th17 subsets, and enhancing T_Reg_ -mediated immunosuppressive functions—may help reestablish immune homeostasis and promote tissue repair (118, 119). Although classical anti-inflammatory agents such as TNF-α inhibitors have already been applied, challenges remain in improving tissue specificity while maintaining the physiological immune functions of macrophages and T cells (120–122). Therefore, identifying more precise immunomodulatory targets holds significant potential for advancing novel therapeutic strategies in chronic wound repair and regeneration.

### 3.4 Immune dysregulation and immune escape in chronic wound healing impairment

During normal wound healing, the immune system precisely modulates the intensity of the inflammatory response and terminates inflammation at an appropriate time (123). However, under conditions of persistent inflammation, immune regulatory mechanisms become dysregulated, and pro-inflammatory cytokines such as IL-1 and IL-6 remain continuously elevated. As a result, the immune response fails to transition in a timely manner from a pro-inflammatory to a reparative state, thereby delaying the healing process (124). Prolonged inflammation may lead to an “overreaction” of the immune system, creating a vicious cycle in which immune cells not only target pathogens but also attack healthy tissues, damaging already repaired structures (68, 125). Although this excessive immune response is widely regarded as a key factor in chronic wound healing impairment, some studies suggest that, in specific contexts, abnormal immune cell activation may not simply represent an “overreaction,” but may instead be closely associated with mechanisms of immune escape (126). Such immune escape may prevent the immune system from effectively clearing local microorganisms or damaged tissue, thereby prolonging the healing process, although its precise molecular mechanisms remain incompletely understood (125). In addition, prolonged exposure of the immune system to a chronic inflammatory milieu may induce a state of “tolerance,” resulting in a marked reduction in immune efficiency and failure to adequately eliminate residual infection or repair tissue damage (127, 128). While this concept is widely supported by multiple studies, other reports indicate that the development of immune tolerance is not the sole contributing factor, and that the interplay and alternation between tolerance and immune escape in chronic inflammation require further distinction and in-depth investigation.

Recent studies indicate that delayed or non-healing chronic wounds are strongly associated with dysregulated adaptive immune responses, particularly the imbalance between effector T cells and T_Reg_. DAMPs, endogenous signals released from injured or necrotic cells, are normally confined to intracellular compartments under physiological conditions. Upon tissue injury, however, DAMPs are released into the extracellular space and activate inflammatory signaling pathways, representing a critical mechanism underlying persistent inflammatory activation and impaired tissue repair in chronic wounds (49, 129). Under normal circumstances, injury-induced stress responses are self-limiting, facilitating macrophage phenotype switching and controlled T-cell activation to promote repair and regeneration. Once the stress resolves, tissue homeostasis is restored (130, 131). In chronic wounds, by contrast, prolonged and amplified stress responses disrupt the balance between effector T cells and T_Reg_, compromise immune tolerance, and sustain chronic inflammation that hinders healing.

When cells are damaged, DAMPs such as high-mobility group box 1 (HMGB1), heat shock proteins (HSPs), ATP, and uric acid are released extracellularly. Elevated circulating DAMPs engage PRRs on dendritic cells and macrophages, thereby activating innate immunity and inducing the secretion of inflammatory mediators including IL-23, IL-17, IL-12, and IL-2 (58, 132). IL-12 and IFN-γ promote CD4^+^ T-cell differentiation toward the Th1 phenotype, while Th1-derived IFN-γ further activates M1 macrophages, reinforcing Th1 responses and suppressing the immunosuppressive function of T_Reg_ (118, 133). This persistent positive feedback loop undermines immune tolerance and sustains a highly pro-inflammatory environment detrimental to wound repair. In chronic wound models, hyperactive Th1 responses and continuous M1 macrophage accumulation are regarded as major barriers to healing (134, 135). Meanwhile, IL-23 and prostaglandin E2 (PGE2) promote Th17, group 3 innate lymphoid cell (ILC3), and γδ T-cell activation, leading to excessive production of IL-17A. IL-17 stimulates epithelial cells, endothelial cells, and fibroblasts to secrete IL-6 and TNF-α, driving the persistent recruitment and aberrant activation of neutrophils and macrophages (136, 137). Overexpression of IL-23 and expansion of Th17 cells further impair T_Reg_ function, aggravating immune imbalance and establishing an irreversible inflammatory state (138). This dysregulation not only disrupts the immune homeostasis required for wound repair but also impedes normal regeneration of fibroblasts and keratinocytes (139).

IL-17A also enhances cyclooxygenase-2 (COX-2)-dependent synthesis of the pro-inflammatory lipid mediator PGE2. Activation of COX-2 and PGE2 promotes vasodilation and leukocyte extravasation, while simultaneously amplifying Th17 activation, thus establishing a positive feedback loop for IL-17A (140, 141). The hyperactive IL-23/IL-17 axis contributes to excessive cytokine deposition within the wound microenvironment, locking the tissue in the inflammatory phase and preventing transition to the proliferative and remodeling phases. Persistent inflammation further induces continuous DAMP release, perpetuating a vicious “injury–inflammation–reinjury” cycle. Accordingly, targeting IL-17 and IL-23—either through specific neutralizing antibodies or in combination with nonsteroidal anti-inflammatory drugs to block the IL-23/IL-17 axis and COX-2 signaling—represents a potential strategy to alleviate chronic wound inflammation (142, 143). In parallel, promoting macrophage repolarization from M1 to M2 phenotypes, restoring T_Reg_ -mediated immunosuppression, and appropriately activating Th2-associated repair pathways could reestablish immune balance, thereby facilitating angiogenesis, cell proliferation, and tissue remodeling to ultimately promote wound healing.

### 3.5 Fibrosis and excessive extracellular matrix synthesis leading to limited angiogenesis

Fibrosis is a process in which tissue repair mechanisms become imbalanced due to repeated injury or chronic inflammation. It is characterized by the excessive accumulation of ECM components, such as collagen, fibronectin, and basement membrane proteins, produced by activated fibroblasts/myofibroblasts. This excess ECM replaces normal tissue structure, leading to tissue stiffening and functional loss (144, 145). During fibrosis, significant changes occur in the mechanical properties of the ECM. For instance, fibrotic ECM exhibits thickened fibers, linearization of collagen fibers, increased cross-linking, and thickening of the basement membrane (146). These changes create a “fibrotic microenvironment,” where the ECM is no longer just a passive structural support but becomes an active “cell regulator” (matrisome) (147, 148). As a result, excessive or abnormal ECM remodeling in chronic wounds or organ fibrosis severely impedes normal angiogenesis during wound healing or tissue repair.

In chronic wounds and organ fibrosis, the excessive deposition and cross-linking of collagen fibers make the ECM stiffer and less deformable. The process of angiogenesis, which involves endothelial cell migration, lumen formation, and basement membrane remodeling, relies on a pliable and elastic ECM scaffold (149). When the ECM becomes too stiff, it inhibits endothelial cell migration and spreading, as cells find it difficult to mechanically sense and compress their way into the matrix. Moreover, a stiffer ECM activates mechanosensitive signaling pathways such as YAP/TAZ, which perpetuate fibroblast activation and lead to the formation of contractile fibrous structures. These structures further compress the space available for angiogenesis. In fibrotic areas, excessive ECM leads to reduced intercellular spacing, ECM thickening, and alterations in the interface between the basement membrane and interstitial matrix, making it difficult for endothelial cells to penetrate or form new capillary beds. Additionally, excessive ECM synthesis often goes hand-in-hand with the persistent activation of pro-fibrotic factors, such as TGF-β, which not only promotes ECM deposition but may also suppress angiogenesis (150). For instance, TGF-β can inhibit endothelial cell proliferation or induce a non-angiogenic state in endothelial cells under certain conditions. ECM itself can bind and store angiogenesis-related growth factors such as VEGF and FGF, but in a fibrotic environment, the release of these factors may be limited (147). High cross-linking of the ECM suppresses the activity of MMPs, enzymes responsible for basement membrane degradation, or stabilizes the ECM structure to the point where it becomes resistant to degradation, thus preventing endothelial cells from forming lumens (151) At the same time, fibrotic regions are often associated with hypoxia or low perfusion, which typically stimulates angiogenesis. However, in highly fibrotic ECM, hypoxia-induced angiogenic signals, such as HIF1α, activate VEGF, which may be “blunted” by mechanical and structural barriers, leading to a weakened angiogenic response (152, 153). Once angiogenesis is impaired, tissue perfusion declines, and the supply of oxygen and nutrients is limited. This, in turn, impacts the cellular metabolism needed for angiogenesis, such as the metabolism of endothelial cells, fibroblasts, and surrounding support cells (154). The decline in mitochondrial function and the increase in ROS, compounded by elevated ECM stiffness, make it difficult for newly formed blood vessels to remain stable (155). Therefore, the “microvascular sparsity and dysfunction” characteristic of fibrotic areas further inhibits vascular regeneration, aggravates tissue ischemia, and impairs tissue repair and remodeling. Recent studies have shown that increased ECM stiffness can activate fibrotic signaling pathways within endothelial cells or surrounding support cells via integrin-dependent mechanisms, indirectly suppressing angiogenesis (156, 157) While much of the research in chronic wound healing has focused on inflammation and immune mechanisms, the changes in ECM density and stiffness caused by fibrosis have also been recognized as key factors in “microvascular generation failure.” Some studies suggest that antifibrotic strategies, such as inhibiting collagen cross-linking or modulating the activity of basement membrane-degrading enzymes, could be new approaches to promoting angiogenesis.

Thus, the excessive synthesis of ECM in fibrosis leading to restricted angiogenesis is a multifaceted process. From mechanical barriers and biochemical microenvironment changes to endothelial cell dysfunction and limited vascular space, as well as metabolic-perfusion deterioration, these factors all contribute to the significant inhibition of new vessel formation. To overcome the problem of delayed microvascular reconstruction in chronic wounds and organ fibrosis, it is necessary not only to regulate immune and inflammatory responses but also to focus on the structural and functional repair of the ECM.

## 4 Biology of inflammation resolution and therapeutic strategies for promoting resolution in chronic wound healing and non-healing delayed wounds

Traditional “anti-inflammatory” approaches primarily focus on inhibiting the inflammatory response itself. In contrast, the biology of inflammation resolution emphasizes that inflammation has an actively programmed “termination mechanism.” Inflammation resolution does not rely on the passive depletion of inflammatory mediators but rather on an actively initiated resolution program. This program’s core tasks include halting the sustained recruitment of granulocytes, clearing dead/damaged cells and necrotic debris, repairing and reconstructing barriers and organ functions, while avoiding an increased susceptibility to infections caused by excessive immune suppression (158, 159). One molecular hallmark of this process is the “lipid mediator switch,” where pro-inflammatory mediators such as prostaglandins and leukotrienes are replaced by specialized pro-resolving mediators (SPMs). These SPMs drive the resolution program without compromising host defense capacity (160, 161). Based on this theory, therapeutic strategies aimed at enhancing immune cell efferocytosis, utilizing IL-10/TGF-β in a context-dependent manner, and targeting key inflammatory pathways can serve as important approaches to treat chronic inflammation and difficult-to-heal wounds.

### 4.1 Direct enhancement of efferocytosis

In various chronic inflammatory diseases, such as atherosclerosis, chronic obstructive pulmonary disease, and inflammatory bowel disease, the clearance of apoptotic neutrophils and foam cells is often impaired. Failure of efferocytosis leads to the expansion of necrotic cores, continued neutrophil infiltration, and compromised repair quality (162, 163). Efficient efferocytosis not only terminates the inflammatory response but also triggers the generation of SPMs, acting as a “final gate” for the resolution of inflammation (164). Therefore, “direct enhancement of efferocytosis” forms a critical foundation for resolution-promoting therapies.

First, efferocytosis can be promoted by releasing “don’t eat me” signals to remove checkpoint inhibition, such as blocking the CD47–SIRPα axis. In a mouse model of atherosclerosis, anti-CD47 antibodies restored efferocytosis within plaques, reduced necrotic core size, and alleviated lesions. The underlying mechanism is closely related to the regulation of the TNF-α–ADAM17 axis (165, 166). CD47–SIRPα-targeting drugs, such as magrolimab and various SIRPα antibodies, have entered multiple clinical trials in oncology; however, known safety risks such as anemia and thrombocytopenia have been identified. The clinical validation of these therapies in chronic injury tissue repair is lacking, indicating that their translation to non-cancer inflammation areas requires careful assessment of the potential benefits and risks (167, 168).

Second, direct activation of phagocytic receptors and their signaling pathways can amplify the efferocytosis response. MerTK, a key phagocytic receptor, enhances macrophage ability to engulf apoptotic cells and promotes SPM synthesis through the nuclear translocation of 5-LOX (169, 170). Studies have shown that MerTK agonistic antibodies have anti-pain and tissue repair-promoting effects in a neuropathic pain animal model, suggesting their cross-organ potential for promoting resolution (171). Further development of TAM receptor-ligand engineering, such as Gas6/Protein S and integrin pathway modulation, could enhance the recognition and engulfment of “Eat-me” signals via biased activation of ligands or antibodies (172). However, it is important to note that excessive Axl activation may promote inflammation or cell migration in certain contexts. Therefore, selective regulation favoring MerTK is preferred (173, 174). Additionally, inhibiting ADAM17/ADAM10-mediated MerTK shedding and maintaining its “grabber” function at the cell surface, or reducing TNF-α stress burden, may help stabilize efferocytosis ability (175, 176).

Third, optimizing the clearance of apoptotic cells by enhancing the recognition and bridging of “Eat-me” signals is another strategy. Recombinant MFG-E8 (lactadherin) can bridge phosphatidylserine (PS) and α_vβ_3/α_vβ_5 integrins, showing potential in preclinical studies of primate periodontal inflammation models, where it inhibits inflammation and preserves bone mass. It is considered a promising local agent for promoting inflammation resolution and wound healing (177).

Overall, direct enhancement of efferocytosis in inflammatory diseases and chronic inflammatory damage is still in the early stages of research. Its organ-specific context dependence and optimal intervention windows need further clarification. When the focus is on clearing large numbers of apoptotic or senescent cells rather than attacking viable tissue cells, de-repression and amplification of efferocytosis can be particularly effective. The key scientific challenge for the clinical application of resolution-promoting therapies is achieving precise and spatiotemporally specific enhancement of efferocytosis without compromising the host’s defense against infection.

### 4.2 Lipid mediator class switch

In the early stages of acute inflammation, pro-inflammatory mediators such as prostaglandins and leukotrienes (e.g., PGE_2_, PGD_2_, LTB_4_) dominate the response. Subsequently, through a programmed “class switch,” the production shifts to specialized SPMs, including lipoxins, resolvins (D/E/n-3 DPA series), protectins, and maresins (159, 178, 179). This transition marks the initiation of the active resolution phase of inflammation, during which granulocyte recruitment is terminated, macrophage efferocytosis is enhanced, and tissue repair and functional reconstruction are activated—rather than the passive “extinction” of inflammation.

In various chronic inflammatory conditions, insufficient production of SPMs, downregulated receptor expression, or impaired intercellular synthesis can lead to a failure of the pro-inflammatory to pro-resolving class switch, resulting in the persistence of chronic inflammation (163). In diseases of the respiratory system, cardiovascular conditions, and immune dysregulation/autoinflammatory diseases, low levels of SPMs or disruption of this “class switch” have been closely linked to increased disease activity and ongoing tissue damage (180).

SPMs initiate a comprehensive resolution program through specific G-protein-coupled receptors such as ALX/FPR2, ChemR23, GPR32, and GPR18, while maintaining the host’s defense capabilities against pathogens (181). This provides a theoretical basis for “supplementing post-switch products,” which can be achieved either by directly supplementing or mimicking SPM analogs, stable derivatives, or through localized delivery systems such as ocular, oral, or pulmonary formulations. In animal models of acute inflammation, low-dose SPM regimens have been shown to significantly shorten the time to inflammation resolution (182). Research on lipid-based resolution mediators has now entered early clinical stages. For example, the novel lipid-derived endogenous molecule RX-10045 (a steroid) has shown good safety in dry eye disease and some efficacy signals, but it has not yet progressed to Phase III clinical trials, indicating that the dosing regimen and pharmacokinetics still require optimization (183). Annexin A1 and its short peptide Ac2–26 exert pro-resolution effects via the ALX/FPR2 receptor, demonstrating protective effects in models of cardiovascular injury and infection, and are actively being evaluated (184). These endogenous resolution pathways can also complement n-3 polyunsaturated fatty acids, which can enhance the levels of SPM precursors and certain SPMs in the body, offering potential to reduce the immunosuppressive side effects of traditional anti-inflammatory drugs while accelerating functional recovery (185, 186).

Overall, the “class switch” of lipid mediators represents a transition from inflammation’s “ignition” phase to “extinguishing and repair.” The success or failure of this switch directly determines whether inflammation can resolve effectively and with high quality. Future pro-resolving therapeutic strategies could involve the use of SPM substitutes/analogs, aspirin- or statin-induced R-pathway activation, enhanced 15-LOX activity, and intercellular synthesis capacity, combined with precursor supplementation and precise local delivery. This approach would transform the intrinsic resolution program into a controllable therapeutic lever. It is important to note that simply inhibiting prostaglandins does not equate to promoting resolution; only when applied at the right time, dose, and target, and combined with quantifiable SPM levels and temporal markers, can “class switch” be effectively controlled and clinically validated.

### 4.3 Targeting inhibition of TLR

The primary function of TLRs is to recognize PAMPs or DAMPs, thereby initiating the innate immune response. In macrophages, TLR signaling preferentially induces the M1 phenotype, promoting high expression of iNOS, TNF-α, IL-6, and IL-1β (187, 188). Furthermore, TLR activation of the NLRP3 inflammasome results in the production of large amounts of IL-1β. IL-1β, through autocrine and paracrine signaling, sustains the M1 phenotype, enhancing glycolytic metabolism and NF-κB signaling activity (189, 190). This persistent pro-inflammatory metabolic state restricts the conversion of macrophages to the M2 phenotype, prolonging the inflammatory process in tissues. In contrast, M2 macrophages are characterized by the secretion of IL-10 and TGF-β and are primarily involved in tissue repair and immune suppression. However, continuous activation of the TLR-NLRP3 pathway can inhibit STAT6 and PPARγ signaling, disrupting the IL-4/IL-13-mediated M2 polarization process. Additionally, IL-1β and IL-18, by promoting IFN-γ and IL-17 production, indirectly hinder the maintenance of the M2 phenotype (188, 189). These mechanisms collectively result in the prolonged dominance of pro-inflammatory M1 macrophages within the immune microenvironment, sustaining the inflammatory response.

TLRs play indispensable roles in innate immunity and the inflammatory response during delayed or non-healing chronic wounds; however, the fine-tuned regulatory mechanisms governing their signaling remain incompletely defined (191, 192). Recent work identifies Cullin 4B (CUL4B), a component of the CUL4B–RING E3 ubiquitin ligase complex, as an intrinsic negative regulator of TLR-triggered inflammation. Loss of CUL4B in macrophages markedly elevates pro-inflammatory mediators (TNF-α, IFN-γ, IL-12, IL-2) while reducing anti-inflammatory factors (TNF-β, IL-10), thereby potentiating inflammation downstream of TLR3, TLR4, or TLR2 stimulation (193). This sustained inflammatory state directly impedes wound repair (**Table 1**).

**Table 1:** Regulatory targets for restoring immune homeostasis, signaling pathways, biological benefits, and preclinical/clinical trials.

Regulatory Type/Regulatory target	Regulatory Methods/Modulator properties	Target properties	Primary target distribution	Main signaling pathways	Biological effects	Preclinical/Clinical Studies	References
Targeting TLR							
CUL4B		Cullin family proteins	T cells, B cells, macrophages,	NF-κB, IRF pathway	Impact on TC and BC activation and function, regulation of immune homeostasis, regulation of cell apoptosis and survival	CUL4B deficiency increases TNF production, reduces IL-10 production, enhances TLR3, TLR4, or TLR2 stimulation intensity, leading to abnormal T cell activation and proliferation	(193)
GSK3β	CHIR99021	Serine/threonine protein kinase	T cells, macrophages	PI3K/Akt, MAPK, NF-κB, Wnt/β-catenin pathway	Inhibition of glycogen synthesis, inhibition of cell proliferation and apoptosis, promotion of TNF-α, IL-1β, and IL-6 expression, promotion of Th17 cell and M1 macrophage polarization	Inhibition of GSK3β activity effectively blocked the increase in TLR-triggered pro-inflammatory cytokine production	(193, 198)
BCAP		PI3K adaptor protein	B cells, myeloid cells	PI3K-AKT, mTOR, NF-κB pathway	B cell development and proliferation, regulation of humoral immune response and inflammatory response, cell survival and metabolic regulation	BCAP-deficient mice exhibit more severe intestinal inflammation	(206)
Targeted NLRP3 Inflammasome							
NLRP3	MCC950	PAMP, NLR family protein	macrophages, neutrophils	NF-κB pathway	Selective inhibition of NLRP3, blockade of inflammasome assembly, reduction of inflammatory cytokine production	Decreased IL-1β production *in vivo*, and alleviation of experimental autoimmune encephalomyelitis	(215, 222)
Targets central cytokines							
TNF-α	Infliximab TNF-α chimeric monoclonal antibody	Pro-inflammatory cytokines	Macrophages, monocytes, T cells	NF-κB, PI3K-Akt, MAPK pathway	Inhibits activation and proliferation of immune cells, reduces the release of inflammatory cytokines and infiltration of inflammatory cells, slows or prevents tissue damage such as joint and intestinal injury	Patients with early active axial SpA receiving combined IFX+NPX treatment are twice as likely to achieve clinical remission compared to those receiving NPX alone.	(202)
	Adalimumab Fully humanized TNF-α monoclonal antibody	Pro-inflammatory cytokines	Macrophages, monocytes, T cells	NF-κB, PI3K-Akt, MAPK pathway	By inhibiting TNF-α, adalimumab reduces inflammation and modulates the immune response	Significantly reduces signs and symptoms in Chinese patients with active AS, improving physical function and quality of life Adalimumab treatment in patients with total spinal ankylosis (TSA) can rapidly improve signs and symptoms of active disease and is clinically significant	(212)
	CT-P13 TNF-α chimeric monoclonal antibody	Pro-inflammatory cytokines	Macrophages, monocytes, T cells	NF-κB, PI3K-Akt, MAPK pathway	CT-P13 binds to TNF-α, preventing it from interacting with its receptors and thereby reducing inflammation	Switching from infliximab to CT-P13 has no negative impact on the safety or efficacy in AS patients CT-P13 has good tolerability, with efficacy and safety comparable to INX	(122) NCT01571206
IL-17	Ixekizumab Humanized IL-17A monoclonal antibody	Pro-inflammatory cytokines	Th17 cells, B cells	NF-κB, MAPK, C/EBP pathway	Inhibits activation and proliferation of Th17 cells, reduces the release of pro-inflammatory cytokines and infiltration of inflammatory cells, slows or prevents joint damage	Superior to placebo in improving radiographic signs and symptoms of axial spondyloarthritis in patients who have not previously received bDMARD treatment	(271)
	bimekizumab IL-17A, IL-17F bispecific monoclonal antibody	Pro-inflammatory cytokines	Th17 cells, B cells	NF-κB, MAPK, C/EBP pathway	IL-17F induces an inflammatory response in skin and joint cells qualitatively similar to IL-17A	Bimekizumab neutralizes IL-17A and IL-17F, more effectively inhibiting *in vitro* cytokine responses and neutrophil chemotaxis	(270)
IL-12/23	Ustekinumab Humanized monoclonal antibody targeting IL-12/23 p40 subunit	Pro-inflammatory cytokines	Dendritic cells, macrophages, T cells	JAK-STAT, NF-κB, MAPK pathway	Inhibits activation and proliferation of Th1 and Th17 cells, reduces release of pro-inflammatory cytokines and infiltration of inflammatory cells, disrupts positive feedback loops	Associated with reduction in signs and symptoms of active AS and good tolerability	(274) NCT01330901
COX	M2000	Nuclear factors, protein kinases, enzymes	T cells, B cells, macrophages	NF-κB, MAPK pathway	Reduced production of TNF-α, IL-1β, and IL-6, decreased activity of T cells and B cells, reduced transcription and expression of NF-kB and COX-2 genes	Compared to placebo, M2000 significantly reduced disease activity in AS individuals, significantly lowering the expression levels of TLR/NF-kB genes M2000 effectively reduced the expression levels of COX-1 and COX-2	(280) (281) IRCT2013062213739N1
	Celecoxib	Enzymes	macrophages, monocytes	NF-κB, PGG2-EP pain signaling pathway	Reduces inflammatory mediators, inhibits prostaglandin synthesis, alleviates pain	NSAID/COX-2 combined inhibitors show significant therapeutic response in Spa	(279)

Augmented TLR-induced inflammation is closely linked to increased glycogen synthase kinase-3β (GSK3β) activity. GSK3β supports prolonged survival and activation of macrophages and lymphocytes, extending the local inflammatory phase and hindering granulation and re-epithelialization (194, 195). The small-molecule inhibitor CHIR99021 binds the ATP site of GSK-3 to inhibit its kinase activity, stabilize β-catenin, and activate Wnt signaling, thereby upregulating repair-associated genes and mitigating chronic inflammation (196, 197). Inhibiting GSK3β effectively lowers TLR-driven TNF-α, IFN-γ, and IL-12 levels and improves the excessive-inflammation–associated mortality observed in Cul4b-deficient mice, suggesting that targeting GSK3β can restrain TLR-related hyperinflammation, restore local immune homeostasis, and promote tissue regeneration and healing (193, 198).

In addition, the TLR signaling adaptor BCAP (B-cell adaptor for PI3K) is critical for limiting overactivation in chronic wound inflammation. Through its YXXM motif, BCAP recruits the p85 subunit of PI3K to activate the PI3K/Akt pathway, indirectly suppressing GSK3β and FOXO1 activity and maintaining immune balance (199). BCAP deficiency reduces PI3K/Akt activity and anti-apoptotic signaling, resulting in persistent activation of inflammatory cells and impaired efferocytosis; increased exposure of self-antigens then drives excessive production of TNF-α, IL-1β, and IL-6, aggravating immune disequilibrium, prolonging the inflammatory phase, and suppressing repair (200–202). Conversely, enhancing BCAP activity may help re-establish local immune homeostasis and a balanced inflammatory milieu, thereby improving chronic wound healing. Notably, because PI3K/Akt signaling can indirectly enhance NF-κB activity, potential adverse effects should be carefully weighed when designing targeted interventions (203, 204).

Aberrant activation of macrophages and T cells is a central mechanism of healing impairment. Persistently activated M1 macrophages release large amounts of pro-inflammatory cytokines and delay the transition to reparative M2 phenotypes; in parallel, abnormal expansion and cytokine secretion by T cells—particularly Th1 and Th17 subsets—amplify inflammation and disrupt fibroblast function and angiogenesis (200, 205). Modulating TLR signaling to recalibrate macrophage and T-cell activation can both attenuate inflammation and restore the function of pro-repair immune populations, thereby accelerating healing in chronic wounds (206).

### 4.4 Targeted inhibition of NLRP3 inflammasome

#### 4.4.1 Targeting NLRP3 mechanisms and promoting chronic wound healing

The IL-1β, IL-6, and IL-23 produced by the NLRP3 inflammasome are key factors in the differentiation of Th17 cells. Initial activation mediated by TLRs creates the necessary inflammatory microenvironment for Th17 differentiation, with IL-1β further enhancing Th17 generation by upregulating the transcription factor RORγt (207, 208). Activated Th17 cells secrete IL-17, which stimulates macrophages and fibroblasts to produce more IL-1β and IL-6, creating a pro-inflammatory positive feedback loop. Additionally, NLRP3 inflammasome activation can induce a shift in T_Reg_ metabolism from oxidative phosphorylation to glycolysis, impairing their immunosuppressive function. This imbalance between Th17 and T_Reg_ cells is a hallmark of chronic inflammatory tissues. The TLR-NLRP3 signaling axis forms a self-amplifying inflammatory circuit: TLR activation upregulates NLRP3 and pro-IL-1β expression (209). Subsequently, NLRP3 inflammasome activation leads to the maturation and secretion of IL-1β and IL-18; IL-1β, in turn, enhances TLR-NF-κB signaling, promoting M1 macrophage polarization and Th17 expansion, which releases more pro-inflammatory mediators like IL-6 and TNF-α (210–212). In this loop, M2 macrophages and T_Reg_ are unable to effectively exert their negative regulatory roles, causing inflammation to transition from an acute response to a persistent chronic state. For instance, in atherosclerosis, TLR4/NLRP3 signaling promotes foam cell formation and exacerbates vascular wall inflammation; in rheumatoid arthritis, the synergistic action of IL-1β and IL-17 amplifies synovial damage (210, 213).

In the delayed healing process of chronic wounds, a persistently activated inflammatory microenvironment is a significant factor hindering tissue repair. As a key regulatory node in the innate immune response, abnormal activation of the NLRP3 inflammasome exacerbates tissue damage and impedes healing. Studies have shown that MCC950, a selective NLRP3 inhibitor, can bind to the NACHT domain of NLRP3, preventing its conformational change and blocking ATPase activity. This significantly reduces the intensity of inflammation and decreases tissue destruction (214, 215). Meanwhile, TLRs, as upstream signaling molecules of the inflammasome, can promote the activation of NLRP3 inflammasomes by initiating the initial signaling, further amplifying the inflammatory response mediated by mature cytokines such as IL-1α, IL-1β, IL-18, and IL-33, thus forming a TLR4/NF-κB/NLRP3 positive feedback loop that delays wound repair (216, 217). In chronic wound tissues, abnormal activation of the NLRP3 inflammasome is often associated with gut microbiota dysbiosis and local immune disturbances, leading to the production of a large amount of type III cytokines and creating an inflammation-resistant environment. MCC950 (also known as CRID3) specifically inhibits the assembly and activation of the NLRP3 inflammasome, significantly reducing the secretion and release of IL-1β and IL-18, weakening chronic inflammation and providing a more favorable immune environment for wound repair (218–220). Notably, MCC950 does not affect the function of other inflammasomes, such as AIM2 and NLRC4, demonstrating its high specificity and clinical application potential. Moreover, immune imbalance in chronic wounds is not only reflected in abnormal inflammasome activation but also involves dysfunction of macrophages and T cells. Over-activated M1 macrophages continue to secrete pro-inflammatory factors, hindering the tissue repair function mediated by M2 macrophages, while excessive activation of effector T cells further amplifies the inflammatory response, resulting in wounds remaining in a prolonged inflammatory state (221). By inhibiting the NLRP3 inflammasome and its associated pathways, the abnormal activity of M1 macrophages and pro-inflammatory T cells can be indirectly reduced, promoting the differentiation of M2 macrophages and the restoration of T_Reg_. This reprogramming of immune cell subsets not only helps alleviate inflammation but also accelerates neovascularization and matrix deposition, thus effectively promoting chronic wound healing (215, 222). In summary, targeting the persistently activated NLRP3 inflammasome can not only relieve excessive inflammation in chronic wounds but also reshape the immune balance of macrophages and T cells, offering a novel therapeutic strategy to enhance tissue repair and regenerate hard-to-heal wounds (221).

#### 4.4.2 Off-target effects of NLRP3 inhibitors

MCC950 is one of the most widely used NLRP3 inflammasome inhibitors, primarily inhibiting inflammasome assembly by blocking the ATPase activity of NLRP3. However, several studies have shown significant off-target effects of MCC950. First, at high concentrations, MCC950 can also inhibit the activation of other inflammasomes, such as NLRC4 and AIM2, suggesting that its action may involve shared regulatory elements, such as NEK7 or mitochondrial ROS (222, 223). Second, MCC950 can interfere with potassium efflux and cell membrane polarization, thus affecting various Ca²^+^-dependent signaling pathways, which is related to its hydrophobic scaffold-mediated membrane binding capacity (224). Moreover, MCC950 has been shown to modulate glycolysis and fatty acid oxidation, which, while helping to reduce NLRP3 activation, may also alter macrophage polarization and T cell metabolic reprogramming, both energy-dependent processes (225, 226). Therefore, the off-target effects of MCC950 are not confined to a single molecular deviation but represent multi-layered interference in inflammatory signaling, membrane electrophysiology, and metabolic reprogramming.

In addition to MCC950, several other NLRP3 inhibitors developed in recent years, such as OLT1177, CY-09, and Oridonin, also exhibit varying degrees of off-target non-specific effects. OLT1177 (dapansutrile) has shown good safety in animal experiments and early clinical trials, but some of its anti-inflammatory effects are related to the inhibition of the IKKβ/NF-κB pathway, not solely dependent on NLRP3 blockade (227). CY-09 competes with ATP by binding to the NACHT domain of NLRP3, but *in vitro* studies have shown that it also inhibits AMPK phosphorylation, suggesting non-specific interference with energy sensing pathways (228). The natural product Oridonin, due to its ability to covalently bind to multiple thiol-containing proteins, not only inhibits NLRP3 but also broadly affects oxidative stress-related signaling and increases cytotoxicity (229). These findings indicate that, even with structural modifications to enhance targeting, small molecules, especially highly reactive natural products, still face significant off-target risks.

The off-target effects of NLRP3 inhibitors present dual impacts in both basic research and clinical translation. On one hand, non-specific inhibition of NF-κB, ROS generation, or metabolic reprogramming may synergistically reduce inflammation, creating an “apparent efficacy” that could be perceived as more effective than actual NLRP3-specific inhibition, thus distorting the accurate interpretation of the mechanism of action (230, 231). On the other hand, such effects may weaken the host’s anti-infection immunity, affect liver drug-metabolizing enzyme activity, induce mitochondrial dysfunction, and even compromise the gut barrier. For patients with chronic diseases requiring long-term medication, prolonged exposure to non-selective inhibitors could potentially induce immune tolerance and metabolic disorders, increasing the risk of adverse effects (226, 232). Therefore, the off-target nature of NLRP3 inhibitors has become a key bottleneck in drug optimization and clinical translation. Future research should combine structural optimization with multi-omics strategies, such as chemical proteomics and single-cell transcriptomics, to systematically map the global effect profile of these inhibitors. Additionally, structural biology and molecular dynamics simulations can be employed to accurately identify critical binding sites and potential non-specific interaction regions. Based on these insights, the development of inducible or tissue-specific NLRP3 inhibition strategies, such as prodrug design, nanoparticle delivery systems, and gene editing regulation, is expected to reduce off-target risks while enabling more precise and safer immune interventions.

### 4.5 Inflammatory microenvironment promotes tissue repair and chronic wound healing

#### 4.5.1 Interactions between macrophages and T cells in the inflammatory microenvironment

At the level of immune regulation, the dynamic transition of macrophage phenotypes is a critical pivot in the progression of a wound from the inflammatory phase to the repair phase. Different T cell subsets finely shape macrophage fate through cytokine feedback loops. IFN-γ, secreted by Th1 cells and CD8^+^ T cells, maintains the inflammatory phenotype of macrophages, prolonging the local pathogen clearance and tissue degradation process in the lesion (233). In contrast, IL-4 and IL-13 produced by Th2 cells drive macrophages to polarize towards a repair-promoting phenotype, facilitating matrix remodeling and angiogenesis (234). IL-17 secreted by Th17 cells enhances neutrophil recruitment and amplifies the inflammatory response, thereby delaying the resolution of inflammation (234). T_Reg_ through the secretion of IL-10, TGF-β, and Amphiregulin, suppress excessive inflammation and directly or indirectly promote tissue repair and regeneration, effectively “braking” macrophage activation (235).

Building on this, the immune checkpoint axis PD-1 (on T cells)–PD-L1 (on macrophages, fibroblasts, neutrophils, and other cells) further limits the function of effector T cells and reversely remodels macrophage transcriptional and metabolic programs (236). The functional effects of this axis vary depending on disease type and the stage of wound healing. In chronic inflammatory environments, overactivation of the PD-1/PD-L1 axis may exacerbate immune suppression and stabilize the inflammatory phenotype (237). In contrast, in the early stages of acute injury, moderate enhancement of this axis helps prevent further tissue damage (238). Therefore, interventions targeting the PD-1/PD-L1 pathway must be precisely aligned with the disease progression and tissue microenvironment. Additionally, macrophages present antigens through MHC-II and co-stimulatory molecules, directly shaping the differentiation profile of CD4^+^ T cells. IFN-γ-induced chemokines such as CXCL9 and CXCL10 continuously recruit effector T cells in chronic wounds, resulting in an “inflammatory residence” state. Single-cell sequencing and spatial omics data indicate that these signaling axes differ significantly between healing wounds and chronic non-healing wounds (239, 240).

Beyond cytokines and receptor signaling, the metabolic microenvironment is another crucial dimension in regulating macrophage–T cell interactions. Hypoxia, high lactate levels, and reprogrammed glucose flux in damaged tissues simultaneously alter the metabolic preferences of both macrophages and T cells. Persistent glycolysis and lactate accumulation not only lock macrophages into an inflammatory phenotype but also impact T cell differentiation and effector function, ultimately creating a “metabolism–immune” coupled pathological loop (241, 242). Breaking this vicious cycle and restoring the spatiotemporal dynamics of macrophage phenotypes could become a key entry point for promoting the transition of chronic inflammation to effective repair in wound healing.

#### 4.5.2 Targeting macrophages and T cells in the inflammatory microenvironment

In delayed or non-healing chronic wounds, neutrophils are among the first immune cells to accumulate at the site of injury, where they generate high levels of ROS via respiratory burst. Excessive ROS not only causes direct tissue damage but also promotes monocyte polarization toward pro-inflammatory M1 macrophages, aberrant activation of effector T cells, and full activation of the NLRP3 inflammasome (243, 244). Together, these events maintain prolonged inflammation in chronic wounds and inhibit tissue repair. Nuclear factor erythroid 2–related factor 2 (Nrf2) is the central transcription factor regulating antioxidant responses. Under oxidative stress, Nrf2 dissociates from its cytoplasmic inhibitor Keap1 and translocates into the nucleus, where it binds antioxidant response elements (AREs) to induce genes such as HO-1 and NQO1. This reduces ROS levels and attenuates inflammation. Studies have shown that dimethyl fumarate activates Nrf2 by covalently modifying Keap1 cysteine residues, while curcumin stabilizes Nrf2 by blocking its ubiquitin-mediated degradation, thereby enhancing both antioxidant and anti-inflammatory effects (245). Moreover, itaconate and its derivative 4-octyl itaconate (4-OI) not only release Nrf2 from Keap1 inhibition but also directly modify NLRP3, reducing excessive inflammasome activation and markedly lowering inflammatory responses in preclinical models (246). These findings suggest that targeting the Nrf2 pathway holds therapeutic potential for chronic wound healing.

From an immunoregulatory perspective, macrophage functional reprogramming is essential for repair. In chronic wounds, excessive M1 activation perpetuates inflammation, whereas M2 macrophages produce pro-repair mediators that promote angiogenesis, collagen deposition, and tissue remodeling (247–249). Thus, inhibiting ROS and NLRP3 inflammasome activity to drive M1-to-M2 repolarization may restore immune balance and accelerate healing. Likewise, effector T cells are often overactivated in chronic wounds, releasing large amounts of inflammatory cytokines (250). Correcting T-cell hyperactivation while enhancing T_Reg_ -mediated immunosuppression can improve the local immune environment and support tissue repair (251, 252).

In addition, extracellular ATP drives membrane depolarization and potassium efflux through P2X7 receptor (P2X7R) activation, a key trigger of NLRP3 inflammasome assembly. Selective P2X7R antagonists such as AZD9056 have shown potential in clinical trials for inflammatory diseases, although with variable efficacy; these results suggest that blocking the ATP–P2X7R axis could represent a viable approach in chronic wound therapy (253). Similarly, NADPH oxidase inhibitors, by reducing ROS production, also demonstrate promise in mitigating chronic inflammation and enhancing repair (254). In summary, targeting ROS and ATP mediated NLRP3 inflammasome activation, together with modulation of macrophage and T cell imbalance, not only alleviates persistent inflammation but also facilitates the transition from the inflammatory to the reparative phase, thereby offering new therapeutic opportunities for refractory wounds.

### 4.6 Contextual utilization of IL-17/TGF-β

#### 4.6.1 Mechanism of contextual utilization of IL-17/TGF-β

IL-17 is an important pro-inflammatory cytokine produced by Th17 cells, γδT cells, and neutrophils. During the early stages of acute wound healing, IL-17 plays a crucial role by recruiting neutrophils, enhancing the expression of antimicrobial peptides, and stimulating fibroblasts to secrete IL-6 and CXCL8, all of which contribute to pathogen clearance and tissue debris removal (255, 256). However, in chronic wounds, the sustained high expression of IL-17 leads to excessive infiltration of inflammatory cells, causing tissue damage and a vicious cycle of oxidative stress.

To address this issue, a key strategy lies in the “temporal regulation” of IL-17. In the early phase of wound healing, moderate activation of IL-17 signaling can enhance immune clearance efficiency. However, in the later stages of wound healing, downregulating IL-17 signaling using TGF-β or exogenous IL-17 receptor antagonists can help resolve inflammation and promote the transition of macrophages from the M1 to the M2 phenotype (257, 258). This “stage-specific utilization—reversal inhibition” strategy demonstrates a contextual approach to managing IL-17, maintaining its immune advantages in the early phase while avoiding its pro-inflammatory side effects in the chronic stage.

TGF-β plays a central role in tissue regeneration and the reversal of inflammation. Through SMAD2/3-mediated transcriptional regulation, TGF-β promotes fibroblast migration and collagen deposition while inhibiting NF-κB signaling and promoting the generation of Foxp3^+^ T_reg_, driving the conversion of inflammation to repair (259, 260). However, in chronic wound models, oxidative stress and a persistent IL-17/TNF-α environment often suppress the action of TGF-β signaling (261). Notably, there exists a feedback relationship between TGF-β and IL-17: TGF-β suppresses the expression of RORγt, inhibiting the differentiation of Th17 cells and thus forming a negative regulatory loop (262).

In the immune system, the interaction between IL-17 and TGF-β is not a simple antagonistic relationship, but rather a bidirectional regulatory mechanism based on the concentration of these cytokines, cell sources, and the timing of signals. Early in inflammation, low concentrations of TGF-β, along with IL-6 and IL-23, coexist to promote the differentiation of Th0 cells to Th17 cells, thereby producing IL-17 to respond to infection (95). Subsequently, sustained TGF-β signaling promotes the generation of T_Reg_ and inhibits Th17 cells, forming a self-limiting inflammatory loop (263, 264). In chronic wounds, restoring the dominant TGF-β signaling pathway becomes crucial for utilizing the endogenous regulatory mechanisms. This approach helps induce M2 macrophages to upregulate IL-10 and Arg-1, clearing residual inflammatory cells and promoting angiogenesis. The transition from IL-17-dominant to TGF-β-dominant signaling forms the molecular basis for inflammation resolution and tissue repair.

#### 4.6.2 Targeting IL-17/TGF-β

Among the pathological drivers of delayed or non-healing chronic wounds, sustained hyperactivation of the IL-23/IL-17 axis is recognized as a major contributor to maintaining the pro-inflammatory microenvironment (264, 265). Within chronic wound tissues, IL-17F plays a pivotal role in promoting neutrophil recruitment and activation, thereby aggravating tissue injury and delaying repair (266, 267). Compared with TNF-α blockade, targeting IL-17A and IL-23 offers higher specificity and efficacy in controlling inflammation (268, 269). Preclinical studies have demonstrated that dual neutralization of IL-17A and IL-17F with antibodies such as bimekizumab significantly reduces cytokine release and neutrophil chemotaxis, thereby attenuating persistent local inflammation and promoting repair (270, 271). Another monoclonal antibody, ustekinumab, simultaneously blocks IL-12 and IL-23 signaling, effectively suppressing Th1 and Th17 overactivation and showing potential to reduce tissue injury and improve reparative conditions in chronic inflammation models (272–274).

**Figure 2:**
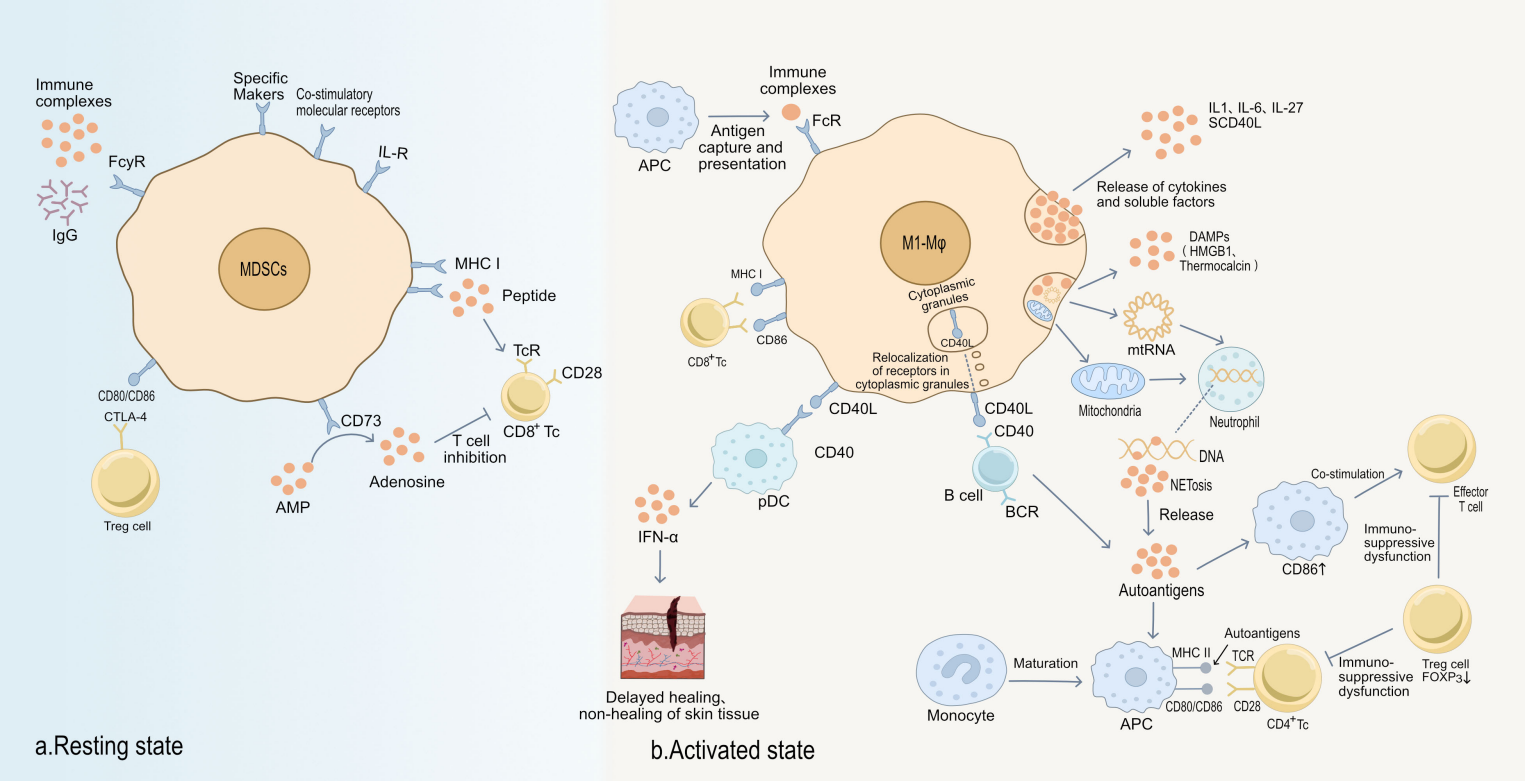
Aberrant APC activation drives delayed or non-healing skin tissue. **(a)** Resting state Tregs express high levels of CTLA-4, which binds to low levels of B7 molecules (CD80/CD86) on APCs. This binding competitively inhibits the CD28-mediated costimulatory signal, thereby limiting the activation of Th cells and CTLs, and maintaining an immune homeostasis conducive to repair. The PD-1 receptor on T cells binds to PD-L1/PD-L2 on APCs, transmitting an inhibitory signal that further suppresses the functions of both T cells and APCs. Additionally, Tregs secrete IL-10 and TGF-β, while MDSCs release ARG1 and iNOS, which directly inhibit the activity of effector T cells. The ectonucleotidases CD39 and CD73 convert ATP/ADP into AMP, which is further converted into adenosine. Adenosine acts on the A2A receptor on T cells, further limiting their activation. In summary, these layered “brake” mechanisms reduce immune-mediated damage to epithelial cells and fibroblasts at the wound edge, preserve the integrity of the extracellular matrix and microvascular network, and support wound healing as planned, rather than allowing the wound to progress to a delayed or non-healing state. **(b)** Activated state. In the “activated state,” abnormally degraded proteins and immune complexes formed by antibodies are recognized through their Fc regions, leading to their uptake, processing, and presentation by APCs, resulting in APC hyperactivation. The upregulation of costimulatory molecules (such as CD40, CD80/CD86, and ICOS) occurs, along with increased secretion of IFN-γ, IL-12, IL-2, and IL-23, which enhances antigen presentation and drives the differentiation of MDSCs and Th0 (CD4^+^ T cells) into M1/Th1/Th2/Th17 subsets. This simultaneously amplifies both CTL-mediated cellular immunity and B cell-mediated humoral immunity. High CD40L expression on Th2 cells further facilitates the full activation of B cells, while IL-2 promotes the proliferation and differentiation of CD8^+^ T cells into effector CTLs. At the same time, the downregulation of negative checkpoint molecules (such as CTLA-4 and PD-1) weakens inhibitory signals, causing Th1, Th2, Th17 cells, and CTLs to become hyperactive and dysregulated. Effector CTLs are subsequently directed to damaged tissues or wounds, where they seek and attack cells bearing self-antigens or damage-associated epitopes. This results in the suppression or killing of epithelial cells and fibroblasts, perpetuating the exposure of self-antigens and the failure to clear them. This leads to a vicious cycle of immune activation and tissue destruction, ultimately resulting in delayed or non-healing of the wound.

Moreover, inflammasome activation in chronic wounds elevates systemic IL-1β and IL-18 levels, which further stimulate IL-23, IL-17A, and IL-22 expression, intensifying local immune imbalance (275, 276). At the same time, cyclooxygenases (COX-1/COX-2), key enzymes in inflammatory signaling, are aberrantly activated in wound tissues, enhancing prostaglandin synthesis and sustaining Th17-driven IL-17A secretion (277, 278). Selective COX-2 inhibitors such as celecoxib partially mitigate chronic inflammation, while novel agents such as β-D-mannuronic acid (M2000) directly bind COX-2, block its catalytic activity, and inhibit NF-κB signaling, thereby improving the local repair microenvironment (279–281). It is important to note that impaired macrophage phenotype switching and T-cell dysfunction also contribute to chronic wound persistence. Under normal conditions, M1 macrophages clear pathogens and necrotic tissue early in healing, before transitioning into M2 phenotypes that promote angiogenesis and matrix remodeling (282, 283). In chronic wounds, however, persistent pro-inflammatory signaling traps macrophages in the M1 state, leading to continuous secretion of IL-1β and TNF-α and perpetuating a deleterious feedback loop. Targeting macrophage polarization to suppress aberrant M1 activation and restore M2 functions could break this inflammatory deadlock and accelerate repair. Similarly, imbalanced CD4^+^ T-cell subsets—particularly hyperactive Th17 cells and insufficient T_Reg_ activity—play a key role in impaired healing. Restoring the Th17/T_Reg_ balance by limiting Th17 expansion and enhancing T_Reg_-mediated immunoregulation represents a promising strategy for chronic wound therapy (**Figure 2**).

Therefore, targeted inhibition of the IL-23/IL-17 axis and related inflammatory pathways, in combination with modulation of macrophage and T cell dysregulation, holds the potential to markedly improve the chronic wound inflammatory microenvironment and facilitate tissue repair. Collectively, these findings highlight the future direction of precision immunomodulation as a cornerstone in chronic wound management.

## 5 Future directions and challenges

### 5.1 Research gaps in the current field

Wound healing is a complex biological process involving multiple layers, such as inflammation, cell migration, angiogenesis, and tissue remodeling. Over the past decades, significant progress has been made in understanding the cellular and molecular mechanisms of healing phases. However, there remains a substantial gap in translating these basic research findings into effective clinical interventions, especially for pathological wounds such as chronic wounds, age-related skin lesions, and diabetes-related ulcers. The limited effectiveness of traditional treatments for these conditions indicates that current understanding of the dynamic changes in the wound microenvironment, immune regulatory networks, and tissue regeneration mechanisms is still insufficient (284). First, incomplete understanding of inflammation and immune response regulation remains a major challenge. The first phase of wound healing—the inflammatory response—plays a central role in activating immune defense mechanisms and initiating the repair response (285). However, current research tends to focus on the relative expression of pro-inflammatory and anti-inflammatory cytokines, lacking a systematic exploration of the “resolution process” of inflammation. For example, the mechanisms of immune cell conversion are insufficiently explored. The phenotypic transitions of macrophages, dendritic cells, and neutrophils at different stages of wound healing are still unclear, and how their plasticity is influenced by local metabolism, mechanical stress, and extracellular vesicle signaling remains to be investigated in greater depth. Furthermore, there is a lack of research on the intersection between immune and metabolic regulation. Recent evidence suggests that metabolic reprogramming directly determines the functional orientation of immune cells (3), yet its role in chronic wound inflammation has not been systematically validated. Second, limitations in studying the microenvironment and ECM signaling hinder further progress. The success of wound healing depends on the coordination between the ECM and intercellular signaling. However, existing research often focuses on isolated signaling pathways (e.g., TGF-β/Smad or VEGF/VEGFR) (286, 287), with insufficient understanding of how multi-signal coupling and physical microenvironmental factors (such as tension, oxygen tension, and ion concentration gradients) work together. For example, there is a lack of integrated studies on the “dynamic microenvironment.” Most experiments are conducted using two-dimensional or static culture models, which fail to reflect the true impact of *in vivo* three-dimensional tension and temporal chemical gradients on cellular behavior. The precise description of ECM remodeling mechanisms is also lacking. The time sequence of matrix degradation and reconstruction, feedback signaling, and their interactions with immune cells have not yet formed a comprehensive theoretical framework.

### 5.2 Future directions and challenges

To address the core issues of sustained inflammation and immune imbalance in wound healing, future research should focus on the dynamic regulation of immune responses and the resolution of inflammation (288). By integrating single-cell transcriptomics, spatial omics, and metabolomics data, we aim to construct spatiotemporal immune maps that reveal the dynamic interactions of macrophages, dendritic cells, and T cells at different stages of healing (289, 290) This approach will provide a molecular foundation for identifying key nodes in inflammation resolution. Moreover, strategies should be developed to precisely intervene in the immune-metabolic coupling, such as using small molecule metabolic regulators or epigenetic modulators to achieve controlled conversion of immune cells from a “pro-inflammatory” to a “repair-promoting” state. The future focus should not solely be on suppressing inflammation, but rather on actively reshaping immune homeostasis, which aims to restore dynamic balance in the immune system without compromising host defense.

Given the complexity and spatiotemporal heterogeneity of the wound microenvironment, traditional two-dimensional models are insufficient for capturing the true biomechanical and signaling gradients. High-fidelity three-dimensional biological response models should be developed, integrating mechanobiology and computational simulations to systematically analyze the combined effects of mechanical stress, oxygen tension, and ion gradients on cellular behavior. In terms of technology, combining spatiotemporal single-cell omics with *in vivo* imaging techniques can enable real-time tracking of the ECM remodeling process, revealing the dynamic regulatory networks involved in matrix degradation and regeneration. The integration of machine learning algorithms to process and analyze these multi-dimensional datasets could help discover new “microenvironmental regulatory factors,” guiding the optimization of biomaterials and drug delivery systems. This could facilitate the shift from descriptive studies to predictive and interventional models.

In the later stages of healing, tissue reconstruction relies on the coordinated action of multiple cell types, including fibroblasts, keratinocytes, endothelial cells, and neurons. Future research should move beyond focusing on individual signaling axes and construct multicellular signaling interaction network models to explore the integrative mechanisms of the neuro-immune-vascular axis in regenerative repair. By employing multi-scale systems biology methods to model the spatial coupling of various signaling pathways, such as TGF-β, VEGF, Notch, and Wnt, we can identify critical regulatory nodes that control the quality of healing (291) This will lay the theoretical foundation for developing comprehensive therapeutic strategies that simultaneously regulate immune responses, angiogenesis, and ECM remodeling.

## 6 Conclusion

Chronic wounds and delayed or non-healing wounds remain significant clinical challenges, and there is currently a lack of drug strategies that can effectively block the sustained activation of the inflammatory response or improve long-term healing outcomes. Just as the intersection of immunology and pharmacology has driven cancer treatment into a new era of “immunopharmacology,” targeting immune imbalance strategies also hold the potential to revolutionize the treatment of chronic non-healing wounds (292, 293). By inducing and maintaining immune homeostasis, it may be possible to more effectively improve the local wound environment, accelerate tissue repair, and ultimately enhance patients’ quality of life and long-term prognosis (294).

Targeting and modulating the chronically activated inflammatory microenvironment to restore immune homeostasis represents an exciting direction for promoting wound healing (295). In chronic wounds, abnormal inflammatory responses driven by both genetic susceptibility and environmental factors often manifest as the persistent overactivation of macrophages and T cells (296). This immune cell dysfunction not only leads to the prolonged secretion of pro-inflammatory cytokines but also suppresses the normal functions of repair-related cells such as fibroblasts, keratinocytes, and endothelial cells, resulting in delayed wound healing (297). Therefore, reprogramming the activity of macrophages and T cells through epigenetic regulation, signaling pathway interventions, and specific inhibitors, to shift them from a “pro-inflammatory” state to a “repair-promoting” state, becomes a central breakthrough for chronic wound treatment.

Although significant progress has been made in understanding how immune cells remodel their function through signaling molecules, several key scientific questions remain to be addressed, including: (1) How do environmental factors in different individuals and populations synergistically influence immune cell activity, and how can we develop precision and personalized therapeutic strategies based on this. (2) How do dynamic changes in epigenetic markers, such as DNA methylation and histone modifications, in the chronic wound inflammatory microenvironment specifically affect gene transcription and cellular function, and how can epigenetic interventions reverse the inflammation-driven wound stagnation. (3) How can we precisely regulate the abnormal activation of macrophages and T cells without weakening normal immune defense.

Furthermore, understanding how upstream immune pathways, such as TCR signaling and cytokine receptors, shape immune cell functional programs may provide new targets for cell-based therapies (298). By precisely modulating these signals, it may be possible to shift the immune microenvironment from a continuously activated inflammatory state to a homeostatic repair state, offering a novel treatment model for chronic wounds (299, 300). These emerging therapeutic strategies not only have the potential to further improve patients’ quality of life but also provide more efficient and personalized solutions for complex immune-mediated non-healing wounds.
